# A Novel Mutation in Thyroid Peroxidase Gene Causing Congenital Goitrous Hypothyroidism in a German-Thai Patient

**DOI:** 10.4274/jcrpe.2503

**Published:** 2016-06-06

**Authors:** Chutintorn Sriphrapradang, Yotsapon Thewjitcharoen, Suwannee Chanprasertyothin, Soontaree Nakasatien, Thep Himathongkam, Objoon Trachoo

**Affiliations:** 1 Mahidol University Faculty of Medicine, Ramathibodi Hospital, Clinic of Medicine, Bangkok, Thailand; 2 Theptarin Hospital, Diabetes and Thyroid Center, Bangkok, Thailand; 3 Mahidol University Faculty of Medicine, Ramathibodi Hospital, Research Center, Bangkok, Thailand; 4 Contributed equally to this work

**Keywords:** thyroid dyshormonogenesis, goiter, thyroid peroxidase, mutation

## Abstract

Thyroid dyshormonogenesis is responsible for 10-15% of all cases of congenital hypothyroidism and is usually inherited. We report a 26-year-old German-Thai male with congenital hypothyroidism caused by a compound heterozygous mutation in the thyroid peroxidase (TPO) gene. He was diagnosed with congenital goitrous hypothyroidism at 4 months of age and had been treated with levothyroxine replacement therapy. His goiter size had increased due to poor compliance to treatment. Ultrasonography of the thyroid gland showed a pattern suspicious for malignancy. The patient later underwent near-total thyroidectomy. Pathologic examination results were consistent with a multinodular goiter and no malignancy. Genetic analyses by direct sequencing of the entire exons and flanking regions of the TPO gene were performed in the index case and family members. The analyses revealed a compound heterozygote of novel TPO mutation of c.1727C>T in exon 10 resulting in amino acid substitution (p.Ala576Val) and c.2268_2269insT in exon 13 causing a frameshift mutation which introduced a stop codon after the insertion site. The latter has been reported in Chinese subjects. However, there is no previous report of c.1727C>T mutation in the literature. We found the allele contained a novel exon 10 mutation inherited from the patient’s German mother and an exon 13 mutation from his Thai father. Analysis using two bioinformatic software programs indicated that this variant was likely to cause damage in the resulting protein molecule. The present report emphasizes the importance of regular follow-up and patient compliance to levothyroxine replacement in patients with goitrous congenital hypothyroidism to avoid prolonged stimulation of thyroid tissue by thyroid-stimulating hormone.

## WHAT IS ALREADY KNOWN ON THIS TOPIC?

The most common defect in thyroid dyshormonogenesis resides in thyroid peroxidase (TPO) gene. The incidence of congenital hypothyroidism due to homozygous TPO defects has been estimated at 1:66,000 for a Dutch population. The salient clinical manifestations of TPO gene mutation are permanent congenital hypothyroidism and goiter, with a variable degree of hypothyroidism and thyroid gland enlargement depending on the severity of the defect.

## WHAT THIS STUDY ADDS?

We report on a novel TPO gene mutation in a German-Thai patient who presented with congenital hypothyroidism and large multinodular goiter. The present report emphasizes the importance of regular follow-up and patient compliance with adequate levothyroxine replacement to avoid prolonged stimulation of thyroid tissue by thyroid-stimulating hormone.

## INTRODUCTION

Congenital hypothyroidism is considered the most common congenital endocrine disorder and causes preventable mental retardation in children with a prevalence of 1 in 2000-4000 live births (1). Thyroid dysgenesis, including agenesis, ectopy and hypoplasia of the gland, is the most frequent cause of congenital hypothyroidism (80-85%); defects in thyroid hormone synthesis (i.e. thyroid dyshormonogenesis) constitute the etiology in the remaining patients. Some clinicians believe that determination of the cause of congenital hypothyroidism is not obligatory due to similar management regardless of etiology. However, to unravel this genetically heterogeneous entity could lead to new possibilities for more specific molecular diagnoses and the discovery of new targets for molecular therapies in the future. Moreover, this knowledge is also useful for providing reliable parental genetic counseling.

Thyroid peroxidase (TPO), an important enzyme in the steps of thyroid hormone synthesis, is located at the apical membrane of thyroid follicular cells. It catalyzes the iodination of tyrosyl residues in thyroglobulin and the coupling of iodotyrosines to produce iodothyronines. Defects in the TPO gene are the cause of the majority of cases of thyroid dyshormonogenesis with permanent congenital hypothyroidism (2,3). Although TPO mutations have been characterized in subjects of various populations in Asia (4,5,6,7,8,9,10,11) including Japanese, Chinese, Malaysian, and Indian, none have been reported in the Thai population to date. Herein, we report on a novel compound heterozygous TPO mutation in a German-Thai patient with permanent congenital hypothyroidism who presented with a huge multinodular goiter necessitating surgical removal.

## CASE REPORT

A 26-year-old man presented with a gradually enlarging multinodular goiter. Previously, he had been a patient in another hospital, but was lost to follow-up in the past 5 years. He had initially presented with delayed bone growth and muscular hypotonia at 4 months of age and was diagnosed to have congenital goitrous hypothyroidism. Levothyroxine (LT4) therapy was started. He was born to non-consanguineous parents. His father is Thai and had no thyroid disorder; however, his mother is German, and in her teens was diagnosed with primary hypothyroidism without goiter and began receiving LT4 replacement. She had neither a history of neck surgery nor radiation. No other family member was reported to have a thyroid disorder.

During childhood, the patient had been regularly followed up by a pediatric endocrinologist. His growth and development were normal except for moderate impairment in fine motor skills and coordination. Ultrasonography of the thyroid gland had revealed that its size was in the upper normal range and LT4 therapy could not be withdrawn. The patient was born and lived in Germany, but later the family moved to Thailand. He graduated with a bachelor’s degree and currently works in the family business. He reported that his goiter size had gradually increased over the past 5 years. He had received LT4 replacement irregularly at a dose of 125 µg/day before he came to us with a concern about the enlargement of his goiter.

At presentation, his body weight was 66 kg and his height was 180 cm. A large multinodular goiter without signs of compression was noted. Thyroid function tests revealed that serum thyroid-stimulating hormone (TSH) level was higher than 100 mIU/L (normal range 0.27-4.2 mIU/L) and serum free thyroxine (fT4) was less than 0.40 ng/dL (normal range 0.93-1.70 ng/dL). Ultrasonography of the thyroid gland showed an enlarged goiter (9.5x8 cm) with multiple solid/cystic nodules in both lobes, ranging in size from 0.8 to 3.6 cm. Microcalcification was also detected in the left lobe of the thyroid gland. At that time, surgery was advised due to a concern about possible thyroid carcinoma. No perchlorate discharge test was done to establish the cause of congenital hypothyroidism. After adequate LT4 replacement, the patient underwent a near-total thyroidectomy and had no complications. Pathology revealed a benign multinodular goiter without any evidence of cancer. Thyroid hormone replacement was given at a dose of 200 µg/day to maintain his thyroid function.

Total triiodothyronine (TT3), fT4, TSH, antibody to TPO (anti-TPO), and antibody to thyroglobulin (anti-Tg) were measured using electrochemiluminescent immunoassays (Abbott Diagnostics, Illinois, USA). Written consent was obtained from the patient and family members. The study was approved by the Ethics Committee of Theptarin Hospital and the Faculty of Medicine Ramathibodi Hospital, Mahidol University.

Genetic analysis was performed in the proband and in all available family members after obtaining informed consent. Because TPO gene mutation is the most frequent cause of thyroid dyshormonogenesis, the entire exons 1-17 and flanking regions of TPO gene were sequenced directly from genomic DNA. To identify a novel mutation, a co-segregation study and human genetic bioinformatics analysis were performed.

**Figure 1:**
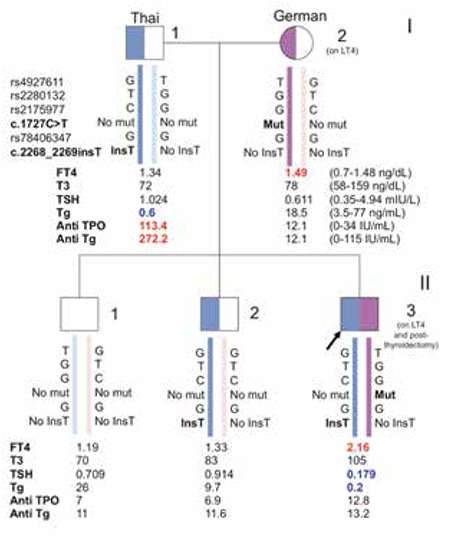
A pedigree of the index patient (II-3) which revealed a compound heterozygote of novel thyroid peroxidase mutation of c.1727C>T in exon 10 resulting in p.Ala576Val and c.2268_2269insT in exon13 causing a frameshift mutation which introduced a stop codon after the insertion site. Exon 10 mutation is maternally-derived, while exon 13 mutation is paternally-derived. The patient’s oldest brother (II-1) had no mutation. Square symbols indicate males, circles females, Roman numerals to the right of the pedigree indicate the generation, and numerals to the right of each symbol indicate individual family members. TSH: thyroid-stimulating hormone, fT4: free thyroxine, T3: triiodothyronine, Tg: thyroglobulin, anti-Tg: antibody thyroglobulin, anti-TPO: antibody thyroid peroxidase

The pedigree is shown in Figure 1. We identified a compound heterozygous mutation on the TPO gene in the index case (II-3). The paternal allele had a frameshift mutation due to an insertion of one nucleotide (c.2268_2269insT) in exon 13. This T insertion caused a stop codon after the insertion point, resulting in a truncated polypeptide of 756 amino acids. The maternal allele had a novel missense mutation (c.1727C>T) in exon 10 resulting in an amino acid substitution from alanine to valine at codon 576 (p.Ala576Val). Based on analysis using two bioinformatic software programs, this variant is likely causing protein damage (SIFT score=0 and Polyphen-2 score=1.0).

These findings were also confirmed by their absence in 100 ethnically matched normal control subjects (courtesy of Professor Joachim Pohlenz).

The patient’s father (I-1) and older brother (II-2) had a heterozygous c.2268_2269insT mutation and normal thyroid function. The father has autoimmune thyroid disease (AITD) as evidenced by high levels of anti-TPO 113 IU/mL (normal range 0-34 IU/mL) and anti-Tg 272 IU/mL (normal range 0-115 IU/mL). The mother (I-2) with primary hypothyroidism was receiving LT4 therapy and had a heterozygous c.1727C>T mutation. She had no positivity of anti-TPO and anti-Tg.

## DISCUSSION

The most common defect in thyroid dyshormonogenesis resides in TPO gene. The incidence of congenital hypothyroidism due to homozygous TPO defects has been estimated at 1:66.000 for a Dutch population (12). The salient clinical manifestations of TPO gene mutation are permanent congenital hypothyroidism and goiter, with a variable degree of hypothyroidism and thyroid gland enlargement depending on the severity of the defect. A severe phenotype resulting in mental retardation and a large goiter has been reported in untreated patients with a complete defect of TPO gene (13). However, some patients who received treatment immediately after birth had normal development without goiter. Also, goiter has been reported to resolve after initiation of LT4 treatment in some patients. In previous studies, enlargement of the thyroid gland was shown in 60-80% of patients, mostly with multinodular appearance, and in some cases with huge goiter or retrosternal invasion necessitating surgical intervention (14). In rare cases, the presence of a thyroid nodule or a goiter in thyroid dyshormonogenesis has been reported to lead to the development of thyroid cancer (15,16). Therefore, in standard practice, all suspected nodules should be evaluated in cases of thyroid dyshormonogenesis.

A delay in treatment of congenital hypothyroidism could partly explain the development of large multinodular goiter in these patients. The diminished thyroid hormone feedback on the pituitary thyrotroph leads to an increase in TSH secretion, stimulating the thyroid gland. Unknown additional factors might also be involved in the development of multinodular goiter, as some patients develop multinodular goiter despite early and adequate LT4 treatment. Organic iodo-compounds have been shown to inhibit thyroid epithelial cell proliferation; therefore, TPO mutations might increase the risk for multinodular goiter due to the lack of these compounds (17). In the present case, the huge goiter most likely resulted from delayed diagnosis and treatment after birth. In addition, poor compliance might have further contributed to goiter enlargement later in life.

Defects in the TPO gene are commonly inherited in an autosomal recessive pattern; therefore, differentiating the genetic basis of congenital hypothyroidism from other causes of hypothyroidism has important implications in terms of genetic counseling. Clinically, a perchlorate discharge test in most TPO gene mutation patients will demonstrate the pattern of total iodide organification defect (18). Unfortunately, our patient did not undergo this test before surgical intervention. In our patient, a compound heterozygous condition of a frameshift mutation from insertion of one nucleotide (c.2268_2269insT) in exon 13 and a novel missense mutation (c.1727C>T) in exon 10 from his mother was confirmed, explaining the molecular basis of the TPO gene mutation. The c.2268_2269insT has been reported in Chinese subjects (5,6). Haplotype analysis revealed that the high prevalence of c.2268insT mutation among Taiwanese is due to a founder effect (6). The ancestors of Taiwanese families have their origins in mainland China. Thailand is home to the largest overseas Chinese community in the world. This could explain why the father of the patient harbored this mutation. However, there is no previous report of a c.1727C>T mutation in the literature. Although functional analysis of missense mutations is important, it is usually not feasible. There are several in silico possibilities to evaluate functional effects of missense mutations. In the present study, analysis using two bioinformatic software programs (SIFT and Polyphen-2) (19,20) demonstrated that this novel mutation is likely to cause protein damage. However, further molecular studies on messenger ribonucleic acid expression might be necessary to help provide a more comprehensive understanding of the exact effect of this novel mutation on the structure and function of the resulting protein. In the largest series of patients with TPO gene mutations (17), a study conducted in Israel, no significant correlation was observed between the specific type of mutation and the severity of clinical presentation. Further case reports for specific mutations should be accumulated in order to gain more detailed insights into the broad phenotypic variations in this entity (21,22).

In summary, we report on a novel TPO gene mutation in a German-Thai patient who presented with congenital hypothyroidism and a large multinodular goiter. The present report emphasizes the importance of regular follow-up and patient compliance to adequate LT4 replacement treatment in patients with goitrous congenital hypothyroidism to avoid prolonged stimulation of thyroid tissue by TSH. There are a small number of previously reported cases of thyroid carcinoma in TPO gene mutation patients who harbored multinodular goiter. Therefore, long-term follow-up of patients with TPO gene mutations is warranted also for early detection of thyroid carcinoma arising in multinodular goiter.

## ACKNOWLEDGMENTS

We would like to thank Dr. Wyn Parksook for his helpful discussion and English editing. We thank Professor Joachim Pohlenz (Johannes Gutenberg University Medical School, Mainz, Germany) for providing the data of German controls. This study was partially supported in grants by the Rare Genetic Disorder Funds, Department of Medicine, Faculty of Medicine Ramathibodi Hospital, Mahidol University.

**Ethics**

Informed Consent: Written informed consent was obtained from the patient and family for the publication of this report and any accompanying images.

Peer-review: External peer-reviewed.

## AUTHORSHIP CONTRIBUTIONS

Concept: Chutintorn Sriphrapradang, Yotsapon Thewjitcharoen, Thep Himathongkam, Design: Chutintorn Sriphrapradang, Yotsapon Thewjitcharoen, Data Collection and/or Processing: Chutintorn Sriphrapradang, Yotsapon Thewjitcharoen, Suwannee Chanprasertyothin, Soontaree Nakasatien, Thep Himathongkam, Analysis and/or Interpretation: Chutintorn Sriphrapradang, Suwannee Chanprasertyothin, Objoon Trachoo, Literature Research: Chutintorn Sriphrapradang, Yotsapon Thewjitcharoen, Writing: Chutintorn Sriphrapradang, Yotsapon Thewjitcharoen.

Financial Disclosure: The authors declared that this study received no financial support.
